# West Nile Virus Infection in Crocodiles

**DOI:** 10.3201/eid0907.020816

**Published:** 2003-07

**Authors:** Amir Steinman, Caroline Banet-Noach, Shlomit Tal, Ohad Levi, Lubov Simanov, Shimon Perk, Mertyn Malkinson, Nahum Shpigel

**Affiliations:** * Hebrew University of Jerusalem Koret School of Veterinary Medicine, Rehovot, Israel; † Kimron Veterinary Institute, Bet Dagan, Israel; ‡ Tel Aviv University Sackler Faculty of Medicine, Tel Aviv, Israel

**To the Editor**: Recently West Nile virus (WNV) infection has been reported in three alligators (*Alligator* sp.) from central Florida (*1*) and one captive crocodile monitor (*Varanus salvadori*) with neurologic signs from the District of Columbia and Maryland area (*2*). These first reports of the virus in American reptiles highlight the possible role of this group of vertebrates in the WNV life cycle. To our knowledge, WNV in a reptile was reported only once before in a serosurvey conducted in Israel from 1965 to 1966, in which 22 reptiles and 96 amphibians were tested for hemagglutination-inhibiting antibodies against several viruses, including WNV; one turtle (*Clemmys caspica*) was seropositive (*3*). Experimental infection of the lake frog (*Rana ridibunda*) with a Russian strain of WNV resulted in high levels of viremia (*4*). At present, the role of reptiles and amphibians in the life cycle and epidemiology of WNV is not known.

We report, for the first time, WNV infection in crocodiles (*Crocodylus niloticus*). To assess the potential role of crocodiles in the life cycle of WNV in Israel, serum specimens were collected from 20 healthy crocodiles on a commercial farm in the Negev Desert, in southern Israel (31°14′N, 34°19′E). The crocodiles came from two separate breeding farms (32°03′N, 35°26′E and 30°18′N, 35°07′E) in the Syrian-African Rift Valley, which is on the main route of bird migration from Africa to Europe. Five males and 15 females, 1–2.5 years of age, were examined. Blood was withdrawn from the crocodiles’ ventral caudal vein, separated by centrifugation, and kept at –20°C until analyzed. Neutralizing antibody titers were determined against WN-goose-98 (*5*) and attempts to isolate the virus were performed by using Vero cell culture (*6*) and by using direct reverse transcription–polymerase chain reaction (RT-PCR) on the serum specimens. To eliminate the possibilities of nonspecific reaction, all serum samples were concurrently tested for the only other flavivirus known to be present in Israel; Israeli turkey meningo-encephalitis virus (ITV) (*7*). Because ITV does not produce cytopathic effects (CPE) in Vero cells, virus neutralization was conducted on BHK cells for both WNV and ITV by using WN-goose-98 and ITV (vaccine strain). In this case, the virus stocks (10^-4.2^ 50% tissue culture infective dose) were diluted 1:400, and virus neutralization titers were checked 3 days later.

Viral RNA was extracted from serum samples with the QIAamp RNA blood kit (QIAGEN, Valencia, CA) , according to the manufacturer’s protocol and resuspended in 30 μl of RNase-free water. The primer pair WN240-Kun848 (respective genome positions 5′: 848 and 1,645) was used to synthesize an 800-bp product in the E gene region (*8*,*9*). The resulting DNA fragment was visualized on 1.5% agarose gel stained with ethidium bromide.

The seroprevalence rate in the first set of virus neutralization assays in Vero cells was 14/20 (70%, with titers ranging from 1:20 to 1:320 [3x1:20, 3x1:40, 3x1:80, 2x1:160, 3x1:320]). No differences were discernible in either the seroprevalence rate or in the average titers of crocodiles from two different breeding farms. In BHK cells, a similar seroprevalence rate was observed, with titers ranging from 1:40 to 1:1,280 (3x1:40, 2x1:80, 1x1:160, 4x1:320, 3x1:640, 1x1:1280). All serum samples, except one, were <1:10 against ITV virus, which had a titer of 1:640 against WNV and 1:10 against ITV. Viremia was not detected in any of the 20 samples in Vero cell culture or by RT-PCR.

These results demonstrate a high rate of infection with WNV in crocodiles in Israel. The crocodiles may have been exposed to the virus during the summer at their present location, since no difference in prevalence was seen between the two groups (which differed only in the farm of origin) and since the younger crocodiles had been hatched in the spring of 2002. Furthermore, a cross-reaction with the other prevalent flavivirus in Israel, ITV, was ruled out. Preliminary results from an equine seroprevalence study (involving 800 horses over a 3-year period) of virus neutralization antibodies to WNV collected during fall 2002, indicate that most horses sampled in Israel’s Arava Valley (a desert in the Syrian-African Rift near the Jordanian and Egyptian borders) and the Gulf of Aqaba/Eilat (30°59′N, 35°18′E to 29°34′N, 34°57′E) (85%, 79/90) were positive (A. Steinman and S. Tal, unpub. data,). WNV was also isolated from mosquitoes in the same region (*10*). The seroprevalence of WNV antibodies among horses and local birds from the Negev Desert is not known nor is the time when the horses acquired WNV infection. However, the isolation of WNV from mosquitoes (*10*) and the presence of antibodies to WNV in young crocodiles demonstrate arboviral activity in this region in the summer of 2002, although clinical cases were few. That virus was not isolated from crocodiles in late November (past outbreaks of WNV in Israel mainly occurred between August and October) (*6*,*11*).

WNV has been endemic in Israel since the early 1950s (*12*). More recently, in the summer of 2000, an extensive outbreak occurred, affecting hundreds of people (*11*), dozens of horses (*6*), and several flocks of geese (*5*). However, no deaths of crocodiles were reported. This contrasts with the report from Florida (*1*), where WNV was isolated from dead alligators, and where hundreds of cases of sudden death had been reported in previous years; these deaths are now suspected to result, at least in part, from WNV disease.

The role of various reptile species in the epidemiology of other arboviruses such as western equine encephalitis, eastern equine ecephalitis, and Venezuelan equine encephalitis is well documented (*13*–*15*). At present, the role of reptiles and amphibians in the life cycle and epidemiology of WNV is not known, and further research is necessary.
